# Diagnostic Utility of Laparoscopy in Breast Cancer-Associated Carcinomatous Peritonitis With Suspected Advanced Ovarian Cancer

**DOI:** 10.7759/cureus.100523

**Published:** 2025-12-31

**Authors:** Toshiya Nishimura, Hideaki Tsuyoshi, Fumikata Hara, Makoto Orisaka, Sayaka Fujiwara

**Affiliations:** 1 Obstetrics and Gynecology, Central Japan International Medical Center, Gifu, JPN; 2 Obstetrics and Gynecology, University of Fukui, Fukui, JPN; 3 Breast Oncology, Aichi Cancer Center Hospital, Nagoya, JPN

**Keywords:** breast cancer, ca15-3, carcinoma of unknown primary origin, carcinomatous peritonitis, diagnostic laparoscopy

## Abstract

Carcinomatous peritonitis (CP) secondary to breast cancer is an uncommon metastatic manifestation that presents considerable diagnostic challenges, particularly when the primary lesion is not detectable through imaging modalities. This report examines the case of a 54-year-old female patient who presented with abdominal pain and constipation. Initial preoperative imaging and tumor marker analysis suggested gynecological malignancy, with a strong suspicion of ovarian cancer; however, no definitive primary tumors were identified. Subsequent diagnostic laparoscopy revealed peritoneal dissemination, and histopathological examination confirmed metastatic breast cancer via immunohistochemical staining. This case highlights the essential role of laparoscopy in obtaining a pathological diagnosis even when gynecological cancer is suspected. Laparoscopy is a minimally invasive and highly effective diagnostic tool for CP, even in cases where the primary breast lesion remains undetected. Early consideration of breast cancer in differential diagnosis may facilitate timely and accurate diagnosis, thereby enabling appropriate management of the condition.

## Introduction

Breast cancer is the most prevalent malignant tumor among women worldwide, with approximately 2,300,000 cases reported by 2022. The age-adjusted incidence rate was approximately 46.8 cases per 100,000 people [1]. The most common sites of distant metastasis include the bone, lungs, liver, and brain, whereas carcinomatosis peritonitis (CP) due to peritoneal dissemination is a relatively rare form of metastasis [2,3].

The diagnosis of this condition is based on clinical symptoms, such as ascites and bowel obstruction, tumor markers (such as CA15-3 and CEA), and imaging studies (computed tomography (CT) and fluorodeoxyglucose F18 (FDG)-positron emission tomography (PET)); however, a definitive diagnosis requires pathological examination.

Laparoscopy has recently gained attention as a minimally invasive diagnostic method, contributing to the improved diagnostic accuracy of peritoneal dissemination in gynecological and gastrointestinal malignancies [4,5]. However, there are few reports on the utility of laparoscopy for breast cancer. In the present case, diagnostic laparoscopy was performed because imaging studies suggested CP of the ovarian cancer. Pathological examination of the resected ovary and peritoneum revealed metastasis from the breast cancer. Although CP due to ovarian cancer was initially suspected, diagnostic laparoscopy revealed ovarian and peritoneal metastases from the breast.

This is a rare reported case in which diagnostic laparoscopy led to the identification of breast cancer metastasis to the ovary and peritoneum, followed by subsequent detection of the primary breast lesion during follow-up.

## Case presentation

A 54-year-old female patient (gravida 3, para 3) presented with abdominal pain and constipation. Her previous intestinal obstruction had initially raised suspicion of transverse colon cancer; however, colonoscopy revealed no abnormalities. Subsequent CT imaging suggested ovarian enlargement, and the patient was therefore referred to our hospital for further evaluation.

The patient measured 157.1 cm in height and weighed 43.2 kg. There was no family history of hereditary cancer. Upon examination following stent placement, the patient's abdominal pain subsided, and no tenderness was detected.

Laboratory investigations indicated a mildly elevated white blood cell count (WBC) of 8340/μL and a C-reactive protein (CRP) level of 0.47 mg/dL. Serum tumor markers, including carcinoembryonic antigen (CEA) at 3.1 ng/mL, carbohydrate antigen 19-9 (CA19-9) at 10 U/mL, and cancer antigen 125 (CA125) at 25.4 U/mL, were all within normal limits (Table 1).

**Table 1:** Laboratory findings of the patient WBC: White blood cells; CRP: C-reactive protein; Hb: Hemoglobin; PLT: Platelet; BUN: Blood urea nitrogen; Cre: Creatinine; AST: Aspartate aminotransferase; ALT: Alanine aminotransferase; LDH: Lactate dehydrogenase; CEA: Carcinoembryonic antigen; CA125: Cancer antigen 125; CA19-9: Carbohydrate antigen 19-9.

Items	Result	Under limit	Upper limit	Unit
WBC	8340	3500	9000	/μL
CRP	0.47	0.00	0.50	mg/dL
Hb	13.0	12.0	16.0	g/dL
PLT	30.3	10.0	40.0	x 10^4^/μL
Na	136.4	136.0	145.0	mEq/L
K	4.2	3.2	5.1	mEq/L
Cl	102.4	98.0	108.0	mEq/L
BUN	12.6	8.0	22.0	mg/dL
Cre	0.65	0.40	0.80	mg/dL
AST	14	8	38	IU/L
ALT	12	4	35	IU/L
LDH	174	119	229	IU/L
CEA	3.1	0.0	5.0	ng/mL
CA19-9	10	0.0	37.0	U/mL
CA125	25.4	0.0	35.0	U/mL

Contrast-enhanced CT of the chest, abdomen, and pelvis revealed enlarged left supraclavicular lymph nodes, which raised suspicion of metastasis from an unknown primary malignancy. No evidence of pulmonary metastases or pleural effusion was observed, and no apparent masses were detected in the breast. Ascites was present in the abdomen, but no significant abnormalities were identified in the pelvic or abdominal organs. Dilatation and fluid retention were noted in the small intestine, suggesting stenosis due to peritoneal dissemination.

**Figure 1:**
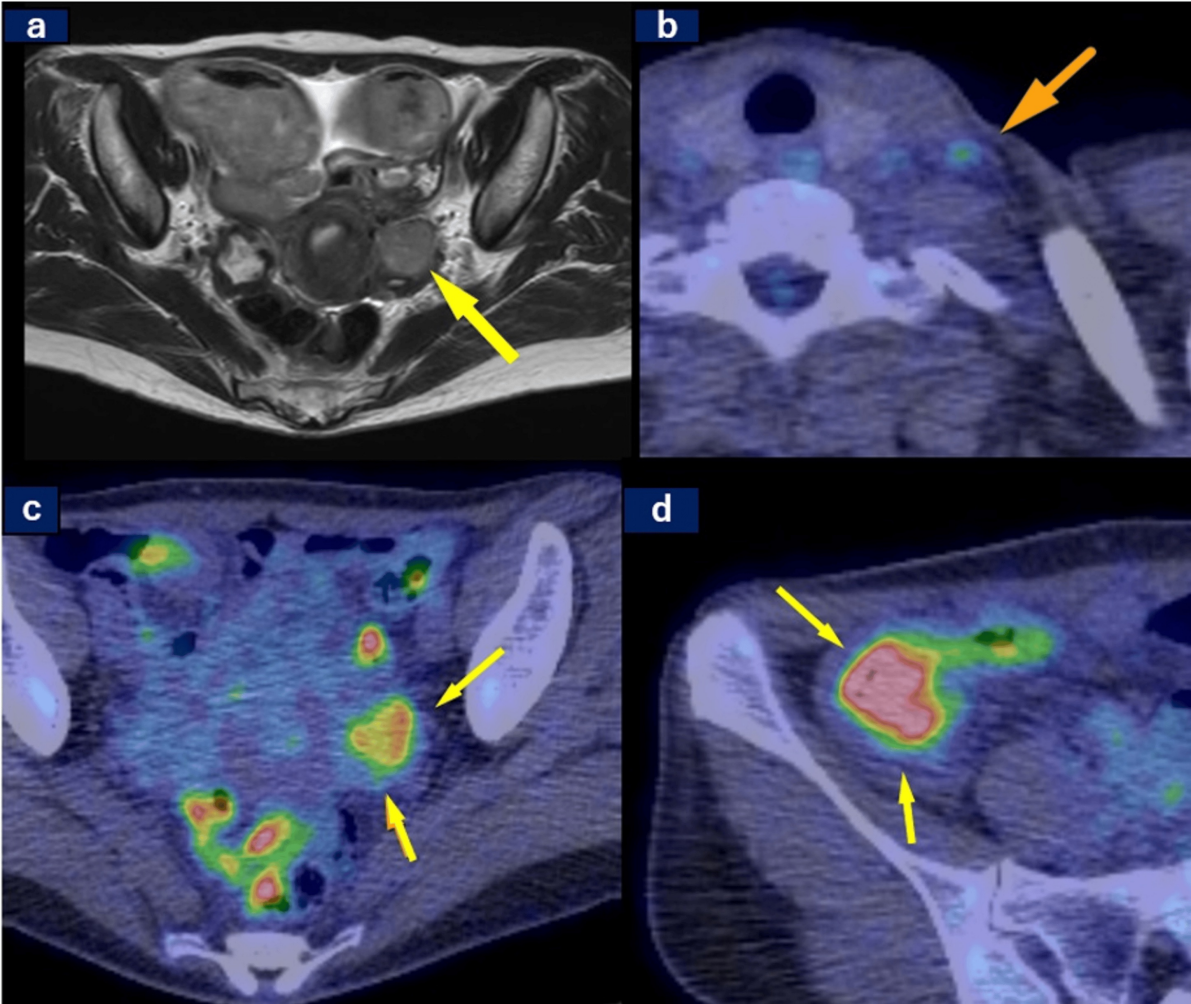
MRI and PET findings: Initially suggested ovarian cancer and cervical lymph node metastasis (a) Magnetic resonance imaging with T2-weighted images showed a left ovarian tumor (arrow). Positron emission tomography (PET) showed the accumulation of fluorodeoxyglucose F18 (FDG) in (b) cervical lymph nodes (arrow), (c) left ovary (arrow), and (d) around the transverse colon stent (arrow).

Pelvic magnetic resonance imaging (MRI) revealed enlargement of the left ovary (Figure 1, Panel a). FDG PET revealed FDG uptake in the left ovary (Figure 1, Panel b), cervical lymph nodes (Figure 1, Panel c), and around the transverse colon stent (Figure 1, Panel d). Based on these findings, ovarian cancer with peritoneal dissemination was considered to be the most probable diagnosis. Diagnostic laparoscopy and cervical lymph node biopsy were performed to obtain tissue samples for pathological confirmation.

**Figure 2:**
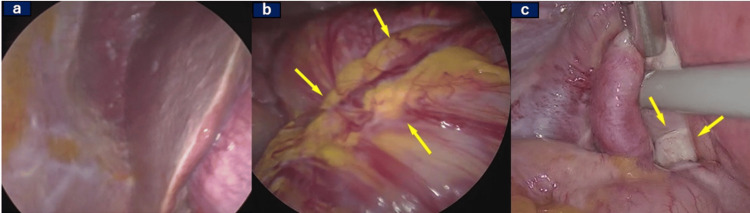
Findings on laparoscopy Laparoscopy revealed (a) miliary-sized peritoneal seeding from the subdiaphragm, (b) the transverse colon was distended due to extensive omental seeding, and (c) no ovarian mass was detected on the left ovary.

Laparoscopic examination revealed miliary-sized peritoneal seeding extending from the subdiaphragm to the Douglas fossa accompanied by ascitic fluid accumulation. The transverse colon exhibited distension due to extensive omental seeding, and the peritoneum surrounding the left ovary was thickened, although no distinct ovarian mass was identified (Figure 2, Panel a). Surgical resection of the omental seeding near the transverse colon (Figure 2, Panel b) and the left adnexa was performed (Figure 2, Panel c).

**Figure 3:**
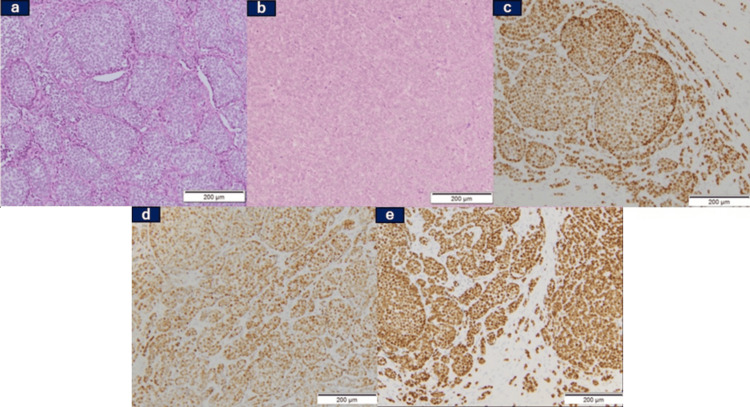
Pathological findings of specimens by laparoscopic examination (a) Hematoxylin and eosin staining revealed poorly differentiated carcinoma, with tumor cells growing within the mesenchyme of the left ovary and the fallopian tube. (b) Cervical lymph node biopsy showing a histological pattern consistent with the peritoneal lesions. Immunohistochemical staining demonstrated positivity for (c) ER(+), (d) PgR(+), and (e) GATA3(+). ER: Estrogen receptor; PgR: Progesterone receptor; GATA3: GATA-binding protein 3.

Histopathological analysis identified a poorly differentiated carcinoma, with tumor cells proliferating within the mesenchyme of the left ovary and fallopian tube (Figure 3, Panel a). Cervical lymph node biopsy revealed a histological pattern consistent with peritoneal lesions, indicating metastasis rather than two separate malignant tumors (Figure 3, Panel b). Immunohistochemical (IHC) analysis revealed positivity for estrogen receptor (ER) (Figure 3, Panel c), progesterone receptor (PgR) (Figure 3, Panel d), and GATA-binding protein 3 (GATA3) (Figure 3, Panel e), suggesting metastatic breast cancer.

**Figure 4:**
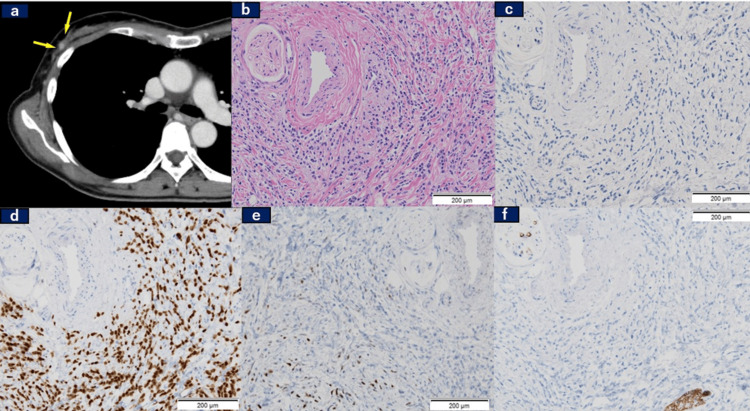
A mammary tumor finally detected 50 days after the initial visit and the associated pathological findings (a) A small nodule emerged. (b) Hematoxylin and eosin staining of the small nodule. Immunohistochemical staining of the small nodule revealed (c) HER2(-), (d) ER(++), (e) PgR (+), and (f) E-cadherin (-). HER2: Human epidermal growth factor receptor 2; ER: Estrogen receptor; PgR: Progesterone receptor.

A follow-up CT scan conducted 50 days after the initial visit to our department revealed a newly detectable mass in region C of the right mammary gland (Figure 4, Panel a), prompting a biopsy from this site. Hematoxylin and eosin (H&E) staining (Figure 4, Panel b) and IHC staining were performed. HER2 staining yielded an IHC score of 0 according to the ASCO/CAP guideline [6], rendering further evaluation using FISH unnecessary (Figure 4, Panel c). Additionally, staining for ER (Figure 4, Panel d) and PgR (Figure 4, Panel e) was positive, and E-cadherin (Figure 4, Panel f) was negative, indicating invasive lobular carcinoma of the breast.

This led to a diagnosis of stage IV breast cancer with peritoneal dissemination, ovarian metastasis, and cervical lymph node metastasis. Subsequently, the patient underwent systemic therapy for metastatic breast cancer. Letrozole was initiated three months after the initial visit, followed by combination therapy with letrozole and abemaciclib after four months. After approximately 13 months of stable disease, follow-up CT demonstrated thickening at the proximal lumen of the transverse colon stent and peritoneal thickening, which was judged to indicate worsening peritoneal seeding; therefore, endocrine therapy was considered no longer effective, and capecitabine was introduced 14 months after the initial presentation, taking into account standard chemotherapy options and the patient’s preference for an oral regimen. At 20 months, CT revealed worsening transverse colon wall thickening and peritoneal thickening with increased ascites accumulation, leading to a determination of progressive disease. As the patient declined further cytotoxic anticancer agents due to concerns about physical decline from side effects and the National Cancer Center (NCC) Oncopanel revealed a high tumor mutational burden (TMB-high), treatment was switched to pembrolizumab monotherapy; however, the patient died 22 months after the initial presentation. Informed consent was obtained for the publication of this case report, and patient anonymity was preserved.

## Discussion

CP originating from breast cancer represents a relatively uncommon metastatic pattern. Bertozzi et al. reported that peritoneal metastases occurred in approximately 0.7% of all patients with invasive breast cancer, including both ductal and lobular subtypes. In their analysis, invasive lobular carcinoma was identified as an independent risk factor for peritoneal metastasis; however, the overall prevalence of breast cancer-related CP remains low, and its prognosis is generally unfavorable [3]. Distinguishing breast cancer with peritoneal dissemination from gynecological or gastrointestinal malignancies is challenging [7]. Pathological examination is crucial for diagnosing breast cancer-related CP alongside imaging findings and tumor marker evaluation, and the diagnostic challenge is exacerbated when the primary lesion is not identifiable.

Diagnostic laparoscopy is well established as an effective tool for assessing peritoneal dissemination in gastric and pancreatic cancers as well as for staging gynecological malignancies [4,5,7]. It has also been reported to be beneficial for confirming the diagnosis and determining treatment strategies in cases of CP in which the primary tumor cannot be clearly identified. However, reports on diagnostic laparoscopy specifically for breast cancer are exceedingly limited, and evidence of its diagnostic utility in this context remains scarce.

Table 1 summarizes previous reports of breast cancer-derived CP along with the present case. The three cases reported by Yoshino et al. [8], Franceschini et al. [9], and Nakagawa et al. [10] involved patients with a history of breast cancer, and CP was confirmed as a recurrence. The case reported by Mitsuyoshi et al. [11] had no prior history of breast cancer but was diagnosed preoperatively based on physical examination and imaging studies. Reports in which breast cancer was not diagnosed preoperatively were limited to cases by Osaku et al. [12] and Saranovic et al. [13]. Osaku et al. [12] described a case of suspected carcinoma of unknown primary origin, where exploratory laparotomy was performed after endoscopic biopsy of a rectal stricture yielded no abnormal findings, and CP due to breast cancer was confirmed through biopsy of the peritoneal implants. Saranovic et al. [13] reported a patient who presented with abdominal distension preoperatively diagnosed with ovarian cancer based on imaging and underwent laparotomy. Postoperative pathology confirmed peritoneal dissemination originating from the breast cancer. In both reports, the diagnosis of breast cancer was established by further breast examination after laparotomy. Egami et al. [14] similarly reported a case in which ovarian enlargement led to a suspicion of ovarian cancer, and peritoneal dissemination from breast cancer was diagnosed laparoscopically, consistent with our case. However, in their case, peritoneal dissemination was limited to the round ligament, and the ovary was diagnosed as a seromucinous borderline tumor. Moreover, no lesions were identified in the breast, which was considered the primary site, even on postoperative imaging; therefore, breast biopsy was not performed.

The patient was initially diagnosed with ovarian cancer due to FDG uptake in the left ovary and subsequently underwent diagnostic laparoscopy for staging purposes. However, intraoperative tissue analysis unexpectedly revealed the presence of metastatic breast cancer. To our knowledge, this is a rare report in which comprehensive preoperative imaging failed to identify the primary lesion, leading to a strong suspicion of ovarian cancer, and in which diagnostic laparoscopy alone resulted in the definitive diagnosis of ovarian metastasis of breast cancer and breast cancer-related CP. Several challenges are encountered during the diagnostic process. FDG-PET demonstrated significant uptake in the left ovary, consistent with ovarian cancer, yet minimal uptake was observed in the pelvic and para-aortic lymph nodes, while the cervical lymph nodes exhibited uptake first, indicating an atypical metastatic pattern for advanced ovarian cancer. Furthermore, the tumor markers CA125 and CA19-9 were within the normal limits, creating a discrepancy with the imaging findings.

As breast cancer was not considered in the preoperative differential diagnosis, the breast cancer-associated marker CA15-3 was not measured. However, as shown in Table 2, several previous reports have documented elevated CA15-3 levels, suggesting its utility as a supplementary diagnostic marker for breast cancer-related CP.

**Table 2:** Case reports of carcinomatous peritonitis in breast cancer CA125: Cancer antigen 125; CA19-9: Carbohydrate antigen 19-9; DOD: Death from disease; AWD: Alive with disease; NA: Not available.

Study (Citation)	Year	Age	Initial/Recurrence	Symptoms	Preoperative Imaging	Tumor Marker (U/mL)	Preoperative Diagnosis of Carcinomatous Peritonitis	Postoperative Diagnosis: Pathological Diagnosis and Subtype (Histological)	Detection of Tumor from the Primary Site (Breast)	Procedure to Confirm the Diagnosis of Peritoneal Dissemination	Outcomes
Yoshino et al. [8]	2024	59	Recurrence	Abdominal distension and ascites effusion	The CT scan showed significant ascites accumulation. There was a partial bowel obstruction.	Not specified	Breast cancer	Same diagnosed from the cell block, we only know it is adenocarcinoma. ER(+), PgR(+), and Her2(−).	Yes (performed total mastectomy before 23 years)	Ascites puncture	DOD (61 months)
Franceschini et al. [9]	2006	67	Recurrence	Abdominal pain associated with constipation, tenesmus, and rectal bleeding	The CT scan showed the pelvic cavity almost completely occupied by neoplastic tissue.	Not specified	Breast cancer	Same invasive lobular carcinoma ER(+), PgR(+), and Her2:NA	Yes	Biopsies taken during rectosigmoidoscopy	AWD (duration not reported)
Nakagawa et al. [10]	2020	68	Recurrence	No symptoms	FDG accumulation was found in the pelvic peritoneum.	CA15-3: 230	Breast cancer	Same invasive ductal carcinoma (papillotubular carcinoma), ER(+), PgR(+), and Her2(−)	Yes	Diagnostic laparoscopy	AWD (duration not reported, chemotherapy was effective)
Mitsuyoshi et al. [11]	2023	48	Initial	A hard mass of 30 mm in size was palpated in area A of the right breast.	FDG accumulation was found in the right breast and pelvic peritoneum.	Not specified	Breast cancer and suspicion of umbilical metastasis	Same invasive ductal carcinoma (tubule forming type + scirrhous type)， ER(+)， PgR(+)，and Her2 (-).	Yes	Diagnostic laparoscopy	AWD （5 months）
Osaku et al. [12]	2015	69	Initial	Constipation	CT and PET did not show any apparent masses in the breasts.	Slightly elevated CA15-3 and CA125	Metastatic disease of any kind	Breast cancer, invasive lobular carcinoma, immunohistochemical findings are NA.	Yes	Diagnostic laparotomy	DOD (48 months)
Saranovic et al. [13]	2011	47	Initial	Abdominal distension, intermittent abdominal pain, and prolonged constipation	CT scan showed peritoneal and omental implants, ascites, and bilateral ovarian cysts. (The breasts are not detailed.)	CA15-3: 66	Ovarian cancer	Breast cancer invasive lobular carcinoma ER(＋), PgR(＋), and Her2 (2+)	Yes	Diagnostic laparotomy	AWD（84 months）, rectal metastasis diagnosed at 72 months
Egami et al. [14]	2024	64	Initial	Back pain	MRI showed 50 mm multicystic tumor on the right ovary.	CA125: 47.2, CA19-9: 1021, and CEA:29.8	Ovarian cancer	Seromucinous borderline tumor. The nodule of the round ligament was adenocarcinoma, and immunohistochemical staining was consistent with breast cancer ER(-), PgR(＋), and Her2 (2+)	No postoperative breast MRI exhibited any malignant findings	Diagnostic laparoscopy	AWD (17 months after operation, chemotherapy was performed)
Our case	2024	54	Initial	Abdominal pain, ileus of the intestine	CT and PET did not show any apparent masses in the breasts.	CA125: 25.4, CA19-9: 10	Ovarian cancer	Breast cancer invasive lobular carcinoma ER(＋), PgR(＋), and Her2:(-)	Yes	Diagnostic laparoscopy	DOD (22months)

## Conclusions

In cases presenting with atypical findings, such as discrepancies between imaging findings and tumor markers, it may be beneficial to include breast cancer in the differential diagnosis, even in the absence of clear breast lesions. Measuring CA15-3, along with a broader panel of tumor markers, may facilitate earlier consideration of breast cancer as a potential origin.

This case highlights the diagnostic challenges associated with occult primary breast cancers. Even when imaging strongly suggests ovarian cancer, clinicians should exercise caution and expand the differential diagnosis when atypical metastatic patterns or inconsistent test results are encountered.
